# Study of the protrusion of through-silicon vias in dual annealing-CMP processes for 3D integration

**DOI:** 10.1038/s41378-024-00797-z

**Published:** 2025-02-13

**Authors:** Tianjian Liu, Shizhao Wang, Fang Dong, Yang Xi, Yunpeng Zhang, Tao He, Xiang Sun, Sheng Liu

**Affiliations:** 1 School of Mechanical Science and Engineering, Huazhong University of Science and Technology, Wuhan, China; 2 School of Power and Mechanical Engineering, Wuhan University, Wuhan, China; 3 The Institute of Technological Sciences, Wuhan University, Wuhan, 430072 China

**Keywords:** Electrical and electronic engineering, Physics

## Abstract

The technology of through-silicon via (TSV) is extensively employed for achieving dense 3D integration. TSV facilitates the electrical interconnection of various layers of integrated circuits in a vertical orientation, thereby allowing for the creation of sophisticated and space-efficient systems that incorporate diverse functionalities. This work reports TSV fabrication with dual annealing-CMP processes to explore the influence of annealing and CMP processes on the evolution of TSV-Cu microstructures and protrusions. The results show that the dual CMP process can effectively reduce protrusion at high temperatures. The Cu protrusion height increased as both the annealing temperature and duration increased, which was consistent with the high-temperature annealing results, whereas a random phenomenon occurred under 250 °C annealing. A phase field model related to the TSV grain size was established to quantitatively explore the grain morphology distribution and thermal-mechanical behavior. The results show that the strain in copper is nonuniform and that the degree of plastic deformation for each grain is closely related to its distribution. The quantity of grains within the TSV is the most important factor for protrusion. As the average grain size increases, the prominence of copper grain protrusions within TSV intensifies, and the anisotropy of the Cu grains becomes more pronounced. The thermal-mechanical behavior strongly depends on the grain orientation near the top of the TSV, which can cause TSV protrusion irregularities. This work may provide more opportunities to design high-performance TSV preparation methods from the viewpoint of the dual CMP process.

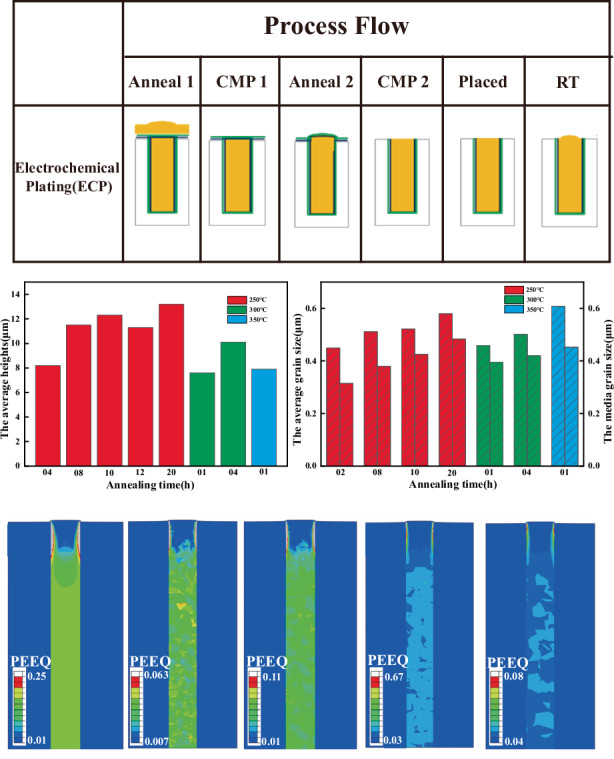

## Introduction

Through-silicon via (TSV) technology plays a crucial role in three-dimensional microelectronic packaging^1^, which has been widely used to realize the interconnection of chips in the stacking direction and is essential for achieving high-density integration. The fabrication of TSV within wafers involves a series of six critical steps, each of which is vital to the process. These steps include the precise creation of vias through deep reactive ion etching, the application of a dielectric layer utilizing plasma-enhanced chemical vapor deposition, the application of barrier and seed layers utilizing physical vapor deposition, and subsequent copper filling via electrochemical deposition. Excess copper is then carefully removed through chemical‒mechanical polishing (CMP). To increase the structural integrity and performance of a wafer, an annealing step is necessary to refine the microstructure, increase ductility, reduce internal stress and lower the resistance of the filled TSV.

Cu serves as a widely adopted filler material in TSV technology but has many reliability issues^2^. In particular, the detailed characterization of the TSV-Cu microstructure is crucial when the thermally induced height increase in TSVs, commonly referred to as “protrusion”, are considered. Cu protrusion leads to further mechanical reliability, such as cracking and delamination of the dielectric layer^3^. The TSV-Cu structure exhibits a considerable coefficient of thermal expansion (CTE) disparity, with a copper CTE of 16.7 ppm/°C, which contrasts sharply with a SiO_2_ of 0.7 ppm/°C and a Si of 2.6 ppm/°C in the surrounding matrix, causing significant stress and deformation under thermal stress. Owing to the high aspect ratio of TSV-Cu, deformation under thermal conditions is generated mainly in the radial direction, which manifests as TSV-Cu protrusions. Although thermal stress is inevitable, we can clarify the mechanical properties by quantitatively analyzing the deformation behavior via analytical or numerical models^4^. Cu protrusion is related to many factors, such as the material process, structure, initial microstructure, initial stress state, and annealing method^5,6^. Typically, an increase in the annealing temperature and duration corresponds to a proportional increase in the height of the protrusions^7,8^. In addition, a reduction in the initial grain size, an increase in the diameter, the presence of a redistribution layer on top, a change in the filling material, or a pre-annealing-CMP treatment can alleviate Cu protrusion^9^. Extensive efforts focused on the design and processing of 3D interconnects with various TSVs have been reported recently, but in-depth studies on new reliability issues related to annealing and CMPs in TSVs are still limited.

While experimental methods such as micro-Raman spectroscopy^10^ and synchrotron micro-beam X-ray diffraction^11,12^ have been employed to evaluate stress, these techniques do not allow for full-field and real-time stress measurements during thermal cycling. Most studies focus on the measurement of protrusions^13^ and microstructural changes after various annealing processes^14^. Accordingly, several methods, such as pre-CMP using or capping layer deposition, are being examined to avoid protrusion^15^. Currently, most studies^16,17^ focus on the thermal stress‒strain distribution caused by thermal mismatch. However, with the rapid development of 3D integration technology, the TSV size has been greatly reduced, which has caused the grain size of the filling material to be comparable to the TSV diameter^18,19^. The thermal-mechanical behavior of TSV is affected by the morphology, orientation and distribution of the grains^20^. In addition, the microstructure evolution and cavity formation in TSV-Cu also affect the thermal-mechanical distribution under annealing and thermal cycling^21^. Although some experimental studies^22,23^ have shown that the stress distribution correlates with the Cu microstructure, quantitative analysis of the effect of microstructure on thermomechanical behavior is lacking. In addition, some simulation studies^24–26^ confirmed that the orientation of the grains does have an effect on the thermal stress distribution on the basis of the assumption of linear elasticity, without considering the plastic deformation of the grains, especially the evolution of the microstructure.

As indicated above, the understanding of the protrusion mechanism remains incomplete and, in some cases, presents inconsistencies. The deformation characteristics of TSV-Cu pose the most significant challenge to the widespread implementation of TSV structures, which requires more detailed study and improvement. In this work, TSV fabrication with dual annealing-CMP processes is proposed, and the influence of the annealing and CMP processes on the evolution of TSV-Cu microstructures and protrusions is explored.

## Materials and methods

**Fig. 1:**
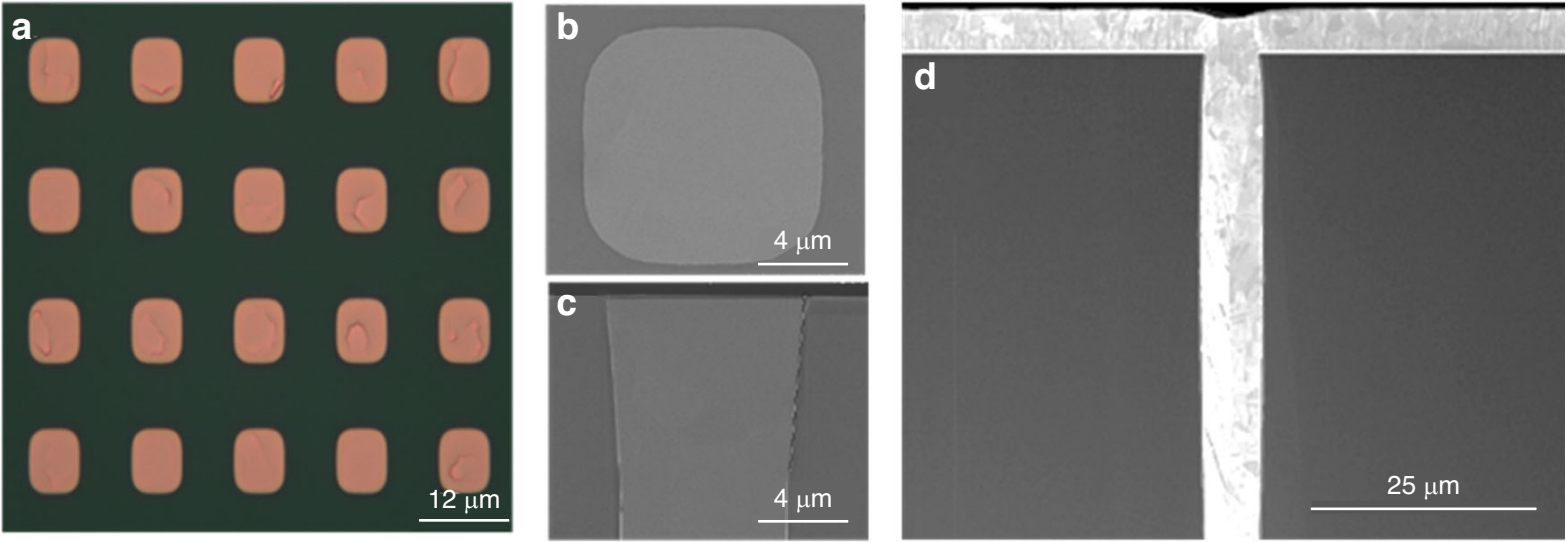
Illustration of the examined samples. **a** Top view of an array of Cu-TSVs, **b** top view of one TSV, **c** part of the TSV cross section, and **d** full TSV cross section

The TSV samples utilized in this study were fabricated on 300 mm silicon wafers with a TSV height of 55 μm and a ratio of ~8:1. A 0.2 μm-thick insulating layer of SiO_2_ was deposited onto the sidewall, followed by the deposition of a 0.01 μm-thick barrier layer composed of Ta/TaN. Figure 1a shows the top view of an array of Cu-TSVs, and Fig. 1b-d shows the top view and cross-section of one TSV, respectively. The copper is free to expand or contract because there is no capping layer on the top of the TSV.

**Fig. 2:**
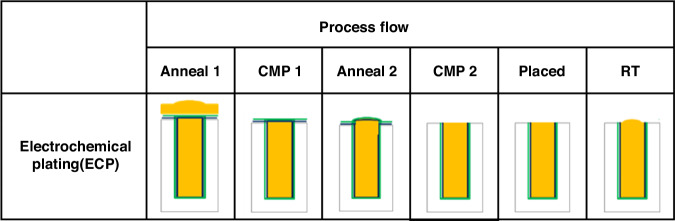
Dual annealing-CMP process flow

The dual annealing-CMP process describes a sequential procedure in which, after copper electroplating for TSV filling, the structure is subjected to an initial annealing treatment, followed by a CMP step; this is followed by a second annealing cycle and another round of CMP processing, as illustrated in Fig. 2.

Considering the temperature range (300–400 °C) for subsequent processes after TSV in the typical wafer manufacturing process and the decreasing tolerance of devices to high temperatures as the processing technology is scaled down, we designed an annealing temperature sequence of 450 °C, 350 °C, and 250 °C. In addition, to obtain an optimized annealing temperature and time through a comprehensive design, we designed short durations at high temperatures and a progressive range from short to long durations at lower temperatures. The Cu-filled TSV samples were subjected to various annealing temperatures and dwell times to explore the occurrence of Cu protrusions, as detailed in Table 1. The TSV samples underwent thermal cycling, where they were incrementally heated from room temperature to 250 °C, 350 °C, and 400 °C. At each of these peak temperatures, the samples were maintained for durations of 1 h, 4 h, 8 h, 10 h, and 12 h. Following the specified dwell times, the samples were allowed to cool to room temperature. TSV samples were subjected to the CMP process. The samples were named S-anneal temperature-dwell time, similar to S-250-10 (peak temperature of 250 °C and dwell time of 10 h). After polishing, the wafers were cleaned and dried. The annealing process and CMP process referenced above were conducted again. Throughout the entire process, argon gas was used to prevent oxidation.

**Table 1:** Anneal temperatures and dwell times

No.	Count	Process Flow				
		Anneal 1	CMP 1	Anneal 2	CMP2	RT
S-400-01	5	400 °C, 1 h	**√**	**-**	**√**	25 °C
S-3500-1	5	350 °C, 1 h	√	350 °C,1 h	√	
S-350-04	5	350 °C, 4 h	√	350 °C,4 h	√	
S-250-04	5	250 °C, 4 h	**√**	250 °C,4 h	**√**	
S-250-08	5	250 °C, 8 h	**√**	250 °C,8 h	**√**	
S-250-10	5	250 °C,10 h	**√**	250 °C,10 h	**√**	
S-250-12	5	250 °C,12 h	√	250 °C,12 h	√	

An optical microscope (OM) was employed to examine the surface morphology of TSV-Cu both prior to and following the annealing-CMP process, aiding in the detection of local protrusions. The heights of the protrusions were measured via an atomic force microscope (AFM, Bruker Dimension Edge). The upper regions of specific Cu vias, which exhibited local copper protrusions, were examined by scanning electron microscopy (SEM, HITACHI SU8600). Additionally, focused ion beam (FIB, ThermoFisher Helios 5UX) milling was employed to prepare cross-sectional slices of the regions of interest for detailed analysis. Electron backscatter diffraction (EBSD, Bruker Weflash FS) techniques were then applied to explore the underlying causes of the local protrusions, with a particular focus on the crystallographic orientations of the copper grains. In addition, a phase field model related to the TSV grain size was established to quantitatively explore the grain morphology distribution and thermal-mechanical behavior.

## Results and discussion

**Fig. 3:**
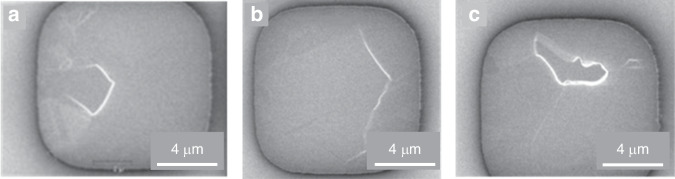
Top-view images of TSVs showing protrusions after various annealing-CMP processes. **a** SEM images of Cu after exposure to 250 °C for 8 h, **b** SEM images of Cu after exposure to 250 °C for 10 h, and **c** SEM images of Cu after exposure to 250 °C for 12 h

Figure 3 shows the TSV protrusion SEM images at 250 °C for 8 h, 250 °C for 10 h, and 250 °C for 12 h after the dual annealing-CMP process. These protrusion characterizations clearly show that plastic deformation occurs in TSV-Cu.

**Fig. 4:**
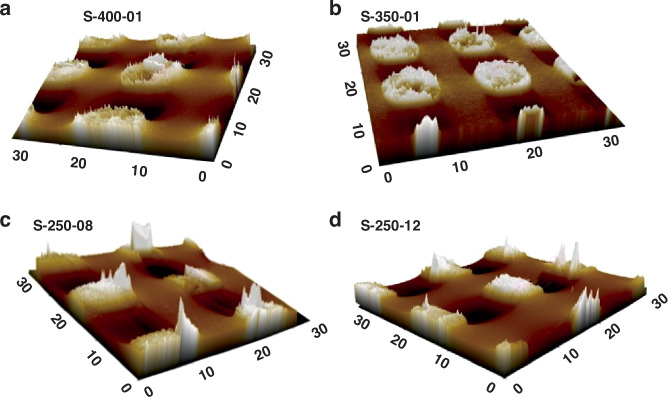
AFM topography measurements of the surfaces of different TSV samples. **a** AFM images of Cu after heating at 400 °C for 1 h, **b** AFM images of Cu after heating at 350 °C for 1 h, **c** AFM images of Cu after heating at 250 °C for 8 h, and **d** AFM images of Cu after heating at 250 °C for 12 h

To compare the extent of Cu protrusion, 3D AFM topographical images under various annealing conditions are shown in Fig. 4. Following the annealing process, the TSV surface exhibited subtle convexity, elevating it above the wafer surface. The irreversible plastic deformation triggered by the thermal expansion of the copper within the TSV is responsible for the residual copper protrusion observed upon returning to room temperature.

To compare the protrusion heights across various samples, we determined the average height values for more than 20 Cu-TSVs. Table 2 shows the change in the average Cu protrusion height with different annealing temperatures and annealing times. Other authors have reported that the protrusion height is positively correlated with elevated annealing temperatures^27,28^, implying that TSV chips operating at higher temperatures are more susceptible to failure^29^.

**Table 2:** Average TSV protrusion heights

No.	Anneal 1	CMP 1	Anneal 2	CMP 2	AFM (Mean)
S-400-01	400 °C,1 h	√	400 °C,1 h	√	7.9 nm
S-350-01	350 °C,1 h	√	350 °C,1 h	√	7.6 nm
S-350-04	350 °C,4 h	√	350 °C,4 h	√	10.1 nm
S-250-04	250 °C,4 h	√	250 °C,4 h	√	8.2 nm
S-250-08	250 °C,8 h	√	250 °C,8 h	√	11.5 nm
S-250-10	250 °C,10 h	√	250 °C,10 h	√	12.3 nm
S-250-12	250 °C,12 h	√	250 °C,12 h	√	11.3 nm
S-250-20	250 °C,20 h	√	250 °C,20 h	√	13.2 nm

By examining and conducting statistical analyses of each sample, it was determined that the local Cu protrusion height of S-400-01 is 7.9 nm, that of S-350-01 is 7.6 nm, that of S-250-08 is 11.5 nm, that of S-250-10 is 12.3 nm and that of S-250-12 is 11.3 nm. These results show that dual annealing-CMP processes can effectively reduce protrusion during high annealing temperatures, thereby improving thermomechanical reliability. Cu protrusion relative to the adjacent material could be observed, and the height increased with increasing peak temperature and total dwell time. During low-temperature annealing, especially at 250 °C, the dual annealing-CMP process led to an increase in the protrusion height, and there was a large difference in the grain size. This phenomenon may have occurred during prolonged 250 °C annealing with the formation of 2 or more nuclei, resulting in the generation of random small grains. Therefore, it can be concluded that the Cu protrusion height of the copper increased progressively with increasing annealing temperature and duration and was highly consistent under high-temperature annealing, whereas a random phenomenon occurred under 250 °C annealing. Long-term annealing at 250 °C should be avoided. In addition, Cu protrusion is related to grain growth (larger grain sizes) and grain sliding caused by stress, whereas recrystallization has the potential to decrease the number of grain boundaries and reduce the excess volume. Therefore, optimizing the Cu grain structure during the electrochemical deposition process and minimizing the residual stress distribution during the annealing process can minimize the Cu protrusion height.

**Fig. 5:**
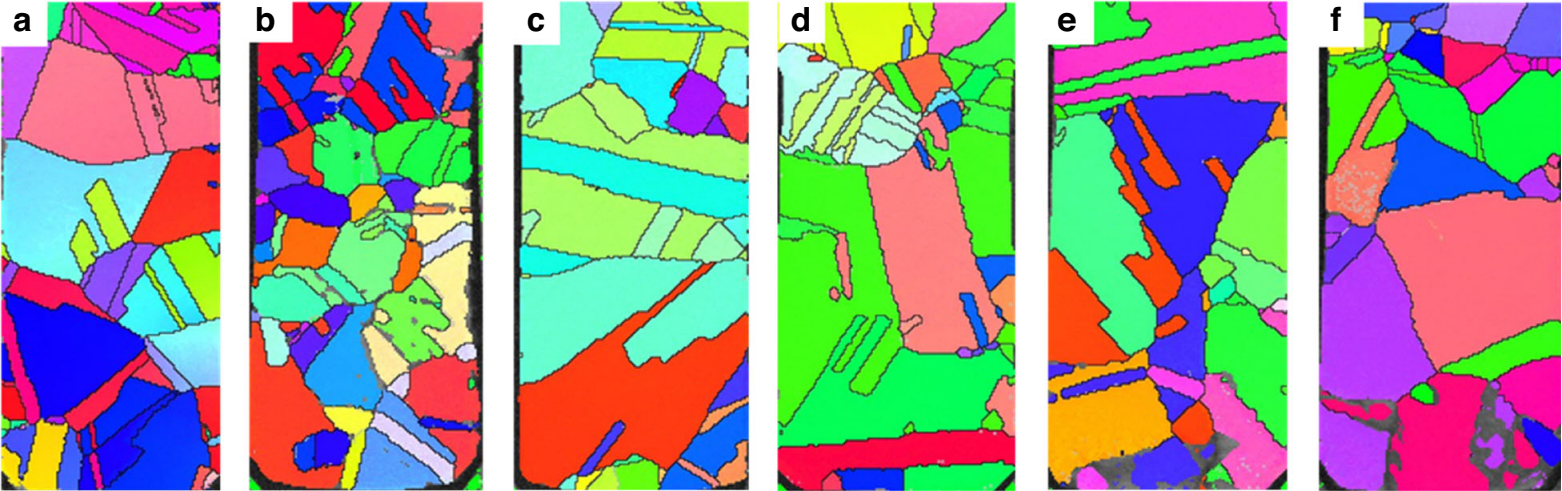
EBSD images of the filled copper in the TSV vertical cross section after dual annealing-CMP processes. **a** 250 °C–8 h, **b** 250 °C–10 h, **c** 250 °C–20 h, **d** 350 °C–1 h, **e** 350 °C–4 h, **f** 400 °C–1 h

**Fig. 6:**
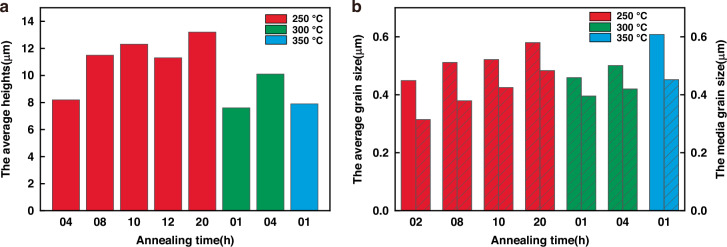
TSV structure changes after annealing. **a** Average TSV protrusion height and **b** average and median grain size distributions

EBSD was employed to analyze the microstructure of TSV-Cu after dual annealing-CMP processes. The average grain size was determined by calculating the mean diameter of the grains within the analyzed region. Figure 5a–e shows EBSD images of a vertical section under different conditions. Post-annealing, there was a notable increase in the grain size, with the majority of the grains evolving into nearly equilateral shapes. Significant grain growth occurred as the residence time increased or the annealing temperature increased. In addition, the correlation between the crystal texture of the TSV filler and the process and geometric parameters cannot be clearly determined. Table 3 presents the distributions of average and median grain sizes following the dual annealing-CMP processes. An increase in the annealing time led to an increase in the Cu grain size. For example, for a sample annealed at 250 °C, the average/median grain size is estimated to be 0.5114 μm/0.3792 μm, and the average/median grain size increases to 0.5799 μm/0.4835 μm after 20 h of annealing, an increase of ~14%/22%. As shown in Fig. 5a–f, when the annealing time is too short, the grains do not have time to fuse and grow, and the grain size distribution is extremely uneven. When the time is too long, the precipitation of crystal nuclei will occur repeatedly to form small grains, which will also lead to an uneven grain size distribution. Both of these factors can lead to inadequate annealing. In addition, the grain distribution during short-term high-temperature annealing has the same effect as that during long-term low-temperature annealing, which enables the growth and redistribution of Cu grains. For example, the average/median grain size is estimated to be 0.50114 μm/0.4201 μm at 350 °C for 1 h and 0.6079 μm/0.4522 μm at 400 °C, an increase of ~21%/7.6%. Therefore, as shown in Fig. 6, an insufficient annealing time or low-temperature annealing hinders grain fusion and growth; thus, extended low-temperature annealing is not recommended. Furthermore, our experiments revealed that annealing at 350 °C for 1 h mitigated the Cu protrusion phenomenon, which may represent optimal process parameters. A comparison of the extent of expansion height with the average grain size reveals that the increase in expansion height does not exhibit a consistent monotonic trend. Hence, the current experimental data do not provide evidence of a direct positive correlation between grain growth and Cu protrusion.

**Table 3:** Average and median grain size distributions after annealing

	250 °C, 2 h	250 °C, 8 h	250 °C, 10 h	250 °C, 20 h	350 °C, 1 h	350 °C, 4 h	400 °C, 1 h
Mean (μm)	0.449	0.5114	0.5215	0.5799	0.4589	0.5011	0.6079
Middle (μm)	0.3142	0.3792	0.4252	0.4835	0.3952	0.4201	0.4522

## Numerical modeling and discussion

Although some studies have confirmed that the grain orientation and distribution in TSV-Cu do affect the thermal stress distribution, the specific influence mechanism is not considered, especially for the microstructure evolution of TSV-Cu under thermal-mechanical loading^25^. Few experimental studies^30^ have reported the effects of microstructure features on Cu behavior in TSVs. The effects of grain shape or other microstructural characteristics on Cu protrusion behavior are not yet systematic or comprehensive; this is mainly because quickly and quantitatively characterizing the influence of microstructural changes via conventional experimental methods is difficult. The finite element method (FEM) may be a good choice for investigating the mechanism of the effect of grain distribution on Cu protrusion.

**Fig. 7:**
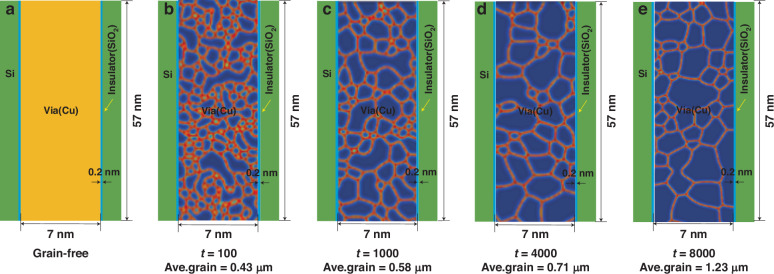
Schematic of a typical Cu-filled TSV structure in a 2D model and grain distributions obtained via phase field modeling for different dimensionless times. **a** Grain-free model, **b** *t* = 100, **c** *t* = 1000, **d** *t* = 4000, and **e** *t* = 8000

To further investigate the protrusion mechanism, the above results are analyzed in depth via the FEM from the perspective of induced grain growth. In this work, Cu-filled TSVs with different grain morphologies were obtained via the phase field method. The influence of the grain morphology and variation in the grain size on the thermomechanical behavior of the samples was explored. The variation in the equivalent plastic strain (PEEQ) with the grain size distribution was also quantified. The typical TSV structure used is shown in Fig. 7, where the gray outline is the Cu grain boundary. The barrier layer materials such as Ta/TaN between Cu and Si are disregarded because of their small thickness. The directional dependence and spatial arrangement attributes of the copper grains were considered, whereas other materials were considered isotropic. To describe the orientation and distribution characteristics of the Cu grains, a set of sequence parameters are introduced: {*θ*_*i*_(*r*, *t*)} (*i* = 1, 2… *N*). The total energy of the polycrystalline TSV-Cu system, characterized by the previously mentioned sequence parameters, can be expressed through the following equation^31^:

1 $$F={{\int}_{V}}\left[\mu {f}_{0}({\theta }_{1},{\theta }_{2},...,{\theta }_{N})+\frac{k}{2}\mathop{\sum }\limits_{i=1}^{N}{(\nabla {\theta }_{i})}^{2}+{E}_{d}\right]{dV}$$

where *k* is the gradient energy coefficient, *u* is a parameter related to the grain boundary energy, and the free energy density function term f can be expressed as:

2 $${f}_{0}({\theta }_{1},{\theta }_{2},...,{\theta }_{N})=\frac{1}{4}+\mathop{\sum }\limits_{i=1}^{N}\left(\beta \frac{{{\theta }_{i}}^{4}}{2}-\lambda \frac{{{\theta }_{i}}^{2}}{2}\right)+\gamma \mathop{\sum }\limits_{i=1}^{N}\mathop{\sum }\limits_{j\ne i}^{N}{{\theta }_{i}}^{2}{{\theta }_{j}}^{2}$$

where *β*, *λ*, *γ* are constants and the minimization of the energy in TSV-Cu drives the movement of the Cu grain boundaries and the evolution of the grain morphology. This dynamic process can be obtained by solving the Allen–Cahn equation as follows:

3 $$\frac{\partial {\theta }_{i}}{\partial \tau }=-\frac{4m}{3{l}_{{GB}}}\frac{\partial F}{\partial {\theta }_{i}}$$

Where *m* is the grain boundary mobility and ${l}_{{GB}}$ is the grain boundary width. The distribution properties and morphological changes of the Cu grains can be dynamically depicted and examined via Eq. 3. Notably, when the above phase field model is used to simulate the morphology characteristics and evolution, the Cu grains in TSV-Cu are assumed to contain only 12 different orientations due to the limited computing resources; that is, 12 Allen–Cahn equations exist.

In this section, the grain morphology in TSV-Cu is constructed based on the Voronoi function via MATLAB software. Notably, through the comparative analysis of previous experimental studies, the assumption of a random distribution of Cu grains is reasonable. The variable *φ* is introduced to visualize the morphology and orientation of the grains, which can be expressed as

4 $$\varphi =\mathop{\sum }\limits_{1}^{12}i{\theta }_{i}^{2}$$

The grain morphology constructed by the Voronoi function serves as the initial value of the sequence parameter in the phase field model, and the grain morphology characteristics can be obtained, as shown in Fig. 7a. To explore the influence of the Cu grain morphology on the thermomechanical behavior, Cu grains with different average sizes were generated and distributed, and the morphologies are shown in Fig. 7b–e). The average grain diameters were 0.43 μm, 0.58 μm, 0.71 μm, and 1.23 μm, respectively. Experimental studies by Chen^32^ et al. also revealed that the diameter of Cu grains in TSV is easily greater than 1 μm. Considering that the diameter can be close to 10 μm, the presence of large grains (or grain coarsening) can lead to a relative decrease in the total number of grains, which will cause changes in the physical properties, especially the anisotropy of the mechanical parameters. In general, grain orientation can be determined by a set of rotation angles in Euler space. For the TSV structure shown in Fig. 7, we randomly set 12 rotation angles corresponding to 12 grains with different orientations. According to the Hall‒Petch law^33^, the yield strength *σ*_*y*_ varies with the grain size, as shown in Eq. 5:

5 $${\sigma }_{y}={\sigma }_{0}+\frac{{k}_{y}}{\sqrt{d}}$$

where *σ*_0_ represents the material constant characterizing the intrinsic stress associated with dislocation motion and where *k*_*y*_ denotes the strengthening coefficient. For Cu in our work, these parameters are 20 MPa and 0.14 MPa m^1/2^, respectively^33^.

**Fig. 8:**
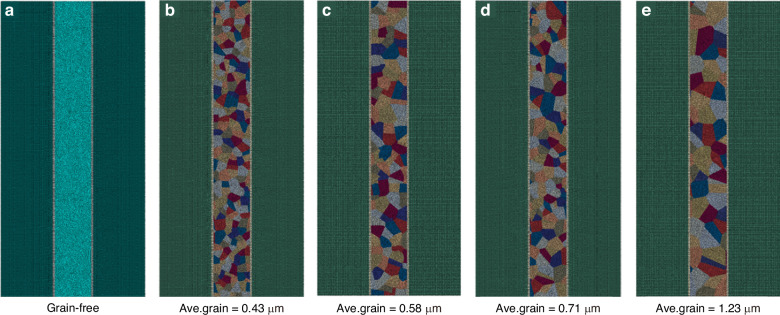
Meshes of different grain distributions in the Cu-filled TSV obtained via phase field modeling for different dimensionless times. **a** Grain-free model, **b** *t* = 100, **c** *t* = 1000, **d** *t* = 4000, and **e** *t* = 8000

Therefore, based on the Hall‒Petch law and the grain size evolution shown in Fig. 8, the yield strengths of different grain distributions can be obtained at 233.5 MPa, 203.83 MPa, 186.15 MPa, and 146.23 MPa. The findings indicate that the yield strengths of the samples prior to annealing fall within the range of 146–235 MPa to 235 MPa, aligning, which aligns closely with the experimental value of 200 MPa reported by Chen et al.^32^. Clearly, the yield strength increases during recrystallization but decreases during grain growth.

To gain insight into the influence of grain size on protrusion, an FEM was established via the commercial software ABAQUS, as shown in Fig. 8, where Fig. 8a) represents the grainless TSV structure and Fig. 8b–e) represent TSV structures with *t* = 100, *t* = 1000, *t* = 4000, and *t* = 8000, respectively. The reference temperature is room temperature, at which all the models are stress-free. The temperature was programmed to increase from room temperature to 350 °C, followed by a decrease to room temperature. Cu is considered an elastoplastic material, with its yield strength assumed to be dependent on the grain size. The mechanical properties of the TSV-Cu used in the model are shown in Tables 4 and 5.

**Table 4:** Young’s modulus and Poisson’s ratio of grains with various orientations

Orientation	Young’s modulus (GPa)			Poisson’s ratio		
	*E*_1_	*E*_2_	*E*_3_	*v*_12_	*v*_23_	*v*_31_
1	152	182	146	0.224	0.313	0.411
2	76	135	136	0.414	−0.051	0.448
3	103	73	107	0.495	0.405	0.166
4	169	100	126	0.480	0.486	0.097
5	94	71	94	0.456	0.418	0.227
6	140	87	149	0.677	0.383	0.001
7	136	79	147	0.495	0.389	−0.061
8	136	71	136	0.493	0.418	−0.095
9	135	144	181	0.441	0.198	0.336
10	156	112	164	0.495	0.352	0.105
11	170	136	138	0.315	0.436	0.240
12	142	125	146	0.418	0.345	0.263

**Table 5:** Elastic material properties of the FEA model

Material	CTE (ppm/°C)	E (GPa)	*v*
Cu	16.7	see Table 4	
Si	020 °C: 2.6 350 °C: 4.4	130	0.28
SiO_2_	0.52	71	0.17

**Fig. 9:**
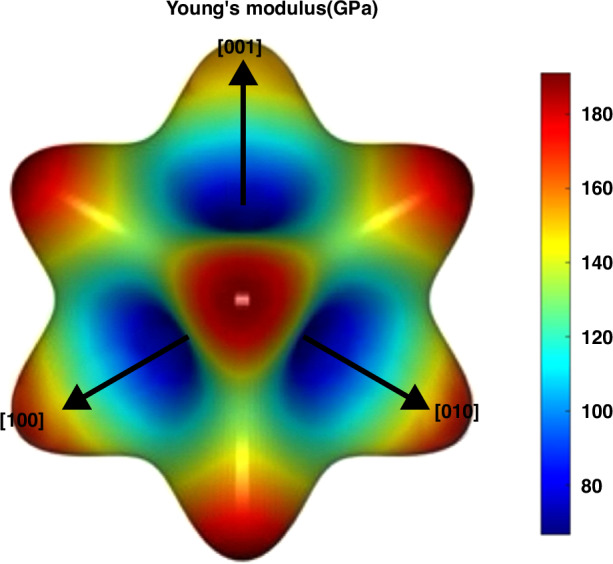
Young’s modulus of different orientations of Cu

The crystal structure of Cu is the face center cubic (FCC), and its elastic stiffness tensor and compliance matrix can be expressed as:

$${\rm{C}}=\left[\begin{array}{cccccc}{{\rm{C}}}_{11} & {{\rm{C}}}_{12} & {{\rm{C}}}_{12} & 0 & 0 & 0\\ & {{\rm{C}}}_{11} & {{\rm{C}}}_{12} & 0 & 0 & 0\\ & & {{\rm{C}}}_{11} & 0 & 0 & 0\\ & & & {{\rm{C}}}_{44} & 0 & 0\\ & & & & {{\rm{C}}}_{44} & 0\\ & & & & & {{\rm{C}}}_{44}\end{array}\right],{\rm{S}}=\left[\begin{array}{cccccc}{{\rm{S}}}_{11} & {{\rm{S}}}_{12} & {{\rm{S}}}_{12} & 0 & 0 & 0\\ & {{\rm{S}}}_{11} & {{\rm{S}}}_{12} & 0 & 0 & 0\\ & & {{\rm{S}}}_{11} & 0 & 0 & 0\\ & & & {{\rm{S}}}_{44} & 0 & 0\\ & & & & {{\rm{S}}}_{44} & 0\\ & & & & & {{\rm{S}}}_{44}\end{array}\right]$$

where C11 = 172.03 GPa, C12 = 121.97 GPa, and C44 = 76.79 GPa. The elastic constant is determined through calculations employing the Vienna Ab initio Simulation Package, with the Perdew–Burke–Ernzerhof generalized gradient approximation selected for the electronic exchange-correlation function^34,35^. The energy cutoff for plane-wave basis expansion was 700 eV. The maximum force tolerance was set to 0.01 eV/Å, and the energy of the self-consistent calculation converged to 10–5 eV. Figure 9 shows the crystal orientation-dependent and anisotropic distributions of Young’s modulus of Cu. In cubic crystal systems, Young’s modulus and the corresponding Poisson’s ratio can be represented by the direction cosines of the three crystallographic directions, [100], [010], and [001]^36^:

$$\frac{1}{{E}_{1}}={S}_{11}({i}_{1}^{4}+{i}_{2}^{4}+{i}_{3}^{4})+({S}_{44}+2{S}_{12})({i}_{1}^{2}{i}_{2}^{2}+{i}_{1}^{2}{i}_{3}^{2}+{i}_{2}^{2}{i}_{3}^{2})$$

$${v}_{{ij}}=-\frac{{S}_{12}+({S}_{11}-{S}_{12}-\frac{1}{2}{S}_{44})({i}_{1}^{2}{j}_{1}^{2}+{i}_{2}^{2}{j}_{2}^{2}+{i}_{3}^{2}{j}_{3}^{2})}{{S}_{11}-2({S}_{11}-{S}_{12}-\frac{1}{2}{S}_{44})({i}_{1}^{2}{i}_{2}^{2}+{i}_{2}^{2}{i}_{3}^{2}+{i}_{3}^{2}{i}_{1}^{2})}$$

$$A=2\frac{{S}_{11}-{S}_{12}}{{S}_{44}},S={C}^{-1}$$

where S11, S12, and S44 represent three independent elastic compliance coefficients. Assume that *i*, *j*, and *k* are the unit vectors orthogonal to each other and that *i*_1_, *i*_2_, and *i*_3_ represent the direction cosines. A is the anisotropic value. Therefore, knowing the values of the three compliance elastic constants, we can obtain Young’s modulus E and Poisson’s ratio *v*. In general, the grain orientation can be determined by a set of rotation angles in three-dimensional Euler space. Given a specified sequence of rotations, where the first rotation occurs by the angle *θ*_*z*_ around the z-axis, followed by a rotation of *θ*_*y*_ around the y-axis, and concluding with a rotation of *θ*_*x*_ around the *x*-axis, the rotation angles are represented by the tuple (*θ*_z_, *θ*_y_, *θ*_x_). By utilizing the specified angles, three rotation matrices, *R*_x_, *R*_y_, and *R*_z_, can be obtained. These matrices, when multiplied, yield the composite direction matrix R’. From this matrix, the rotated unit vectors I’, J’, and K’ can be determined. Similarly, the remaining vectors J and K are calculated via an analogous method. Ultimately, the elastic modulus and Poisson’s ratio for the specified direction are deduced from these rotations and vectors. (See MATLAB code in Appendix A).

$${R}_{x}({\theta }_{x})=\left[\begin{array}{ccc}1 & 0 & 0\\ 0 & \cos {\theta }_{x} & -\sin {\theta }_{x}\\ 0 & \sin {\theta }_{x} & \cos {\theta }_{x}\end{array}\right]$$

$${R}_{y}({\theta }_{y})=\left[\begin{array}{ccc}\cos {\theta }_{y} & 0 & \sin {\theta }_{y}\\ 0 & 1 & 0\\ -\sin {\theta }_{y} & 0 & \cos {\theta }_{y}\end{array}\right]$$

$${R}_{z}({\theta }_{z})=\left[\begin{array}{ccc}\cos {\theta }_{z} & -\sin {\theta }_{z} & 0\\ \sin {\theta }_{z} & \cos {\theta }_{z} & 0\\ 0 & 0 & 1\end{array}\right]$$

$$R^{\prime} ={R}_{z}{R}_{y}{R}_{x}$$

$$I^{\prime} =({R^{\prime} }_{11},{R^{\prime} }_{12},{R^{\prime} }_{13})\,J^{\prime} =({R^{\prime} }_{21},{R^{\prime} }_{22},{R^{\prime} }_{23})\,K^{\prime} =({R^{\prime} }_{31},{R^{\prime} }_{32},{R^{\prime} }_{33})$$

$${i}_{1}=\cos (i,I^{\prime} )\,{i}_{2}=\cos (i,j^{\prime} )\,\,{i}_{3}=\cos (i,K^{\prime} )$$

As shown in Fig. 8, 12 sets of rotation angles corresponding to different crystal orientations were set. In this section, any 12 rotation directions are randomly selected, and the rotation angle sequences are as follows: for the initial six grain orientations, the grains are rotated in the order of the z-axis, y-axis, and x-axis. For the other six grain orientations, the grains are rotated in the order of the z-axis, x-axis, and y-axis. The twelve sets of rotation angles are 45°, 14.5°, 37.5°; 13°, 79°, 142.5°; 153°, 0°, 97.5°; 30°, 146°, 12.5°; 90°, 67°, 0°; 76°, 132°, 153°; 30°, 76.5°, 18°; 45°, 90°, 0°; 132°, 169°, 56°; 95°, 30°, 38°; 60°, 20°, 130°; and 150°, 15°, 35°. Table 4 provides Young’s modulus and Poisson’s ratios for grains with different orientations.

**Fig. 10:**
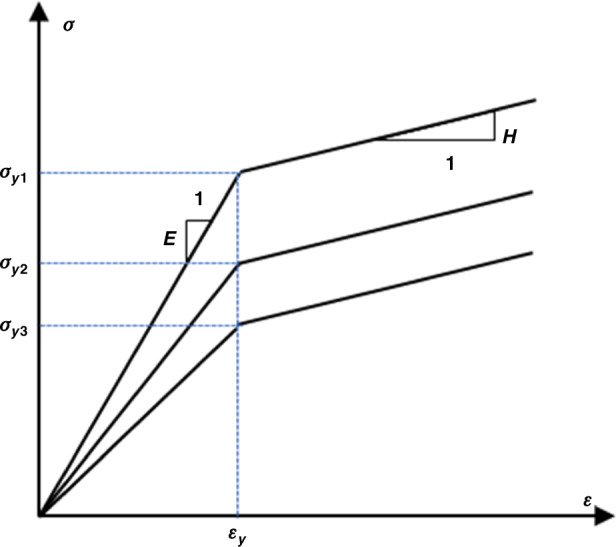
Elastoplastic stress‒strain curve based on the isotropic hardening model

The thermal stress of the TSV structure can be greater than the yield strength under high-temperature loading. The plastic deformation behavior of Cu can be characterized by the linear isotropic hardening model shown in Fig. 10. The stress‒strain relationship can be determined by the yield stress and tangent modulus H.

**Fig. 11:**
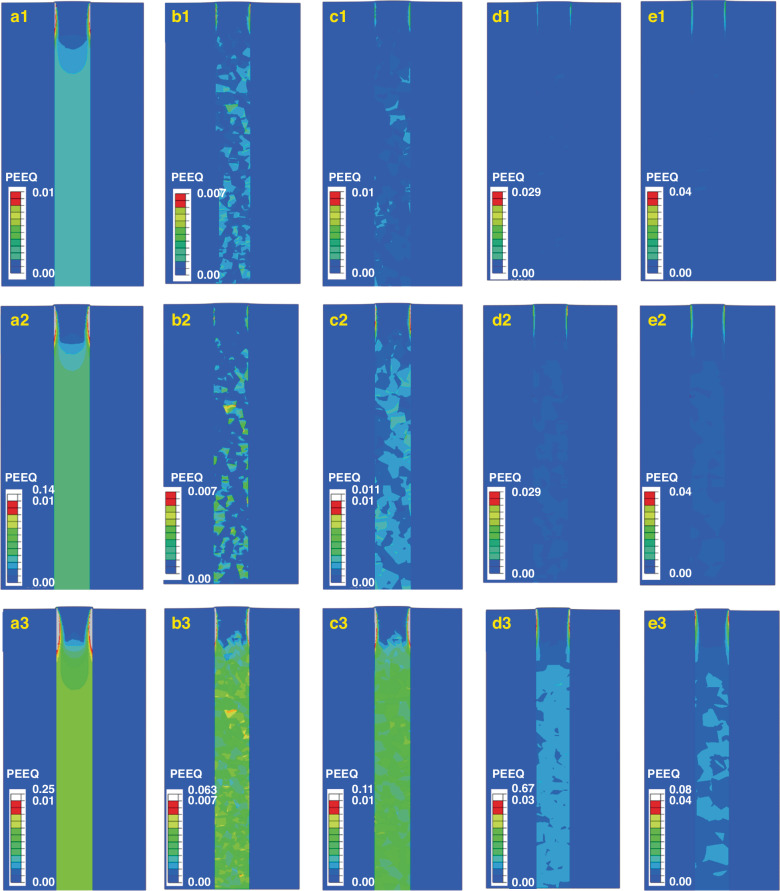
PEEQ distribution. Distributions of PEEQ in the Cu-filled TSV without considering Cu grains (**a1**–**a3**) and with considering Cu grains of different distributions (**b1**–**b3**), (**c1**–**c3**) and (**d1**–**d3**) corresponding to Fig. 7a–d, respectively

Figure 11a shows the PEEQ distribution without considering the influence of the Cu grain morphology, and the PEEQ distributions considering the different Cu grain morphologies are shown in Fig. 11b–e. The strain is clearly symmetrically distributed when the influence of the Cu grain morphology is not considered, as shown in Fig. 11a1-a3. As shown in Fig. 11b–e, the strain distribution is uneven and closely related to the grain distribution characteristics, which is consistent with the results of this experiment.

At 200 °C, plastic changes occur only in a few grains. As the temperature increased to 250 °C, more Cu grains underwent plastic deformation, as shown in Fig. 11b2-e2. As the temperature increased to 350 °C, the majority of the grains experienced plastic deformation, as shown in Fig. 11b3–e3; this is mainly because there are greater stresses on the grain boundaries between different grains. However, the number of grain boundaries in Cu with a small grain size is greater (Fig. 11b1–b3), resulting in a smaller PEEQ. When the Cu grain distribution is considered, the number of grains is limited, and the average PEEQ increases with increasing grain size. The anisotropy in the mechanical properties of the Cu grains is primarily responsible for the finding that the average PEEQ is influenced primarily by the specific orientations of the Cu grains rather than their average size.

### Relationship between the grain size evolution and protrusion

**Fig. 12:**
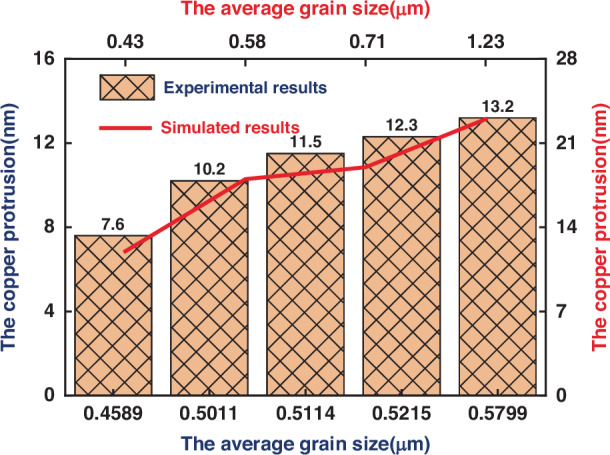
Cu protrusion evolution with respect to the average grain size in the simulation and experimental results

The different grain topographies obtained by the phase field model in the previous section were introduced into the TSV Cu and subsequently annealed at a peak temperature of 350 °C. The protrusion was measured when the temperature decreased to 25 °C. The protrusion evolution curve with respect to the average grain size was obtained, and the protrusion heights at grain sizes of 0.43 μm, 0.58 μm, 0.71 μm, and 1.23 μm were 12 nm, 18 nm, 19 nm, and 23 nm, respectively. As shown in Fig. 12, our simulation results maintain good qualitative consistency with the experimental results; that is, with increasing grain size, the protrusion of Cu becomes more obvious.

These observations indicate that when the grain morphology is considered, the height of the protrusions within the TSV increases alongside the increase in the average grain size. This is because Cu with a small grain size has greater yield strength, and plastic deformation occurs only when it is subjected to higher stress. The yield strength gradually decreases with increasing average grain size, and the copper is more prone to plastic deformation. As a result, the thermal-mechanical behavior is strongly dependent on the grain orientation near the top of the TSV, which causes some TSV protrusions to be irregular.

In addition to the influence of the Cu yield strength or plastic deformation, other factors may also impact the protrusion behavior. TSV-Cu undergoes creep deformation due to annealing at a high temperature, which may cause additional protrusion. During annealing, enhanced shear stresses develop at the interface between TSV-Cu and the silicon substrate, potentially resulting in frictional or atomic diffusion-driven sliding along this interface^37^. This interfacial movement further exacerbates the formation of Cu protrusions.

## Conclusion

The effects of different annealing-CMP processes on the microstructure evolution of TSV-Cu were investigated in this work. The protrusion mechanism was also explored by analyzing the micromorphology evolution under different thermal loading conditions. The results are as follows:

The dual annealing-CMP process can effectively reduce protrusion at high temperatures. Too short of an annealing time or too low of a temperature will cause the grains to have no time to fuse and grow. In addition, if the annealing time is too long, repeated nuclei precipitate to form small grains. Therefore, long-term annealing at low temperatures should be avoided.

A phase field model related to the TSV grain size was established to quantitatively explore the grain morphology distribution and thermal-mechanical behavior. The different grain distributions in TSV-Cu cause the strain to be unevenly distributed, and the degree of plastic deformation for each grain is also closely related to its distribution. The grain count within the TSV is a critical determinant of the protrusion height. When the grain morphology is enhanced, the protrusion height within the TSV increases with increasing average grain size, and the anisotropy of the Cu grains becomes more pronounced. The thermal-mechanical behavior is strongly dependent on the grain orientation near the top of the TSV, which causes some TSV protrusions to be irregular.

## Supplementary information

Appendix A
